# A Survey of MIKC Type MADS-Box Genes in Non-seed Plants: Algae, Bryophytes, Lycophytes and Ferns

**DOI:** 10.3389/fpls.2018.00510

**Published:** 2018-04-18

**Authors:** Gokilavani Thangavel, Saraswati Nayar

**Affiliations:** Division of Plant Molecular Biology, Rajiv Gandhi Centre for Biotechnology, Thiruvananthapuram, India

**Keywords:** MADS box, MIKC type, non-seed plants, evolution, algae, bryophytes, lycophytes, ferns

## Abstract

MADS box transcription factors have been studied extensively in flowering plants but remain less studied in non-seed plants. MADS box is one such example of a gene which is prevalent across many classes of plants ranging from chlorophyta to embryophyta as well as fungi and animals. MADS box transcription factors are of two types, Type I and Type II. Type II transcription factors (TF) that consist of a MADS domain, I region, K domain, and C terminal domain are discussed in this review. The Type II/ MIKC class is widespread across charophytes and all major lineages of land plants but unknown in green and red algae. These transcription factors have been implicated in floral development in seed plants and thus the question arises, “What is their role in non-seed plants?” From the studies reviewed here it can be gathered that unlike seed plants, MIKC^C^ genes in non-seed plants have roles in both gametophytic and sporophytic generations and contribute to the development of both vegetative and reproductive structures. On the other hand as previously observed in seed plants, MIKC^*^ genes of non-seed plants have a conserved role during gametophyte development. With respect to evolution of MIKC genes in non-seed plants, the number of common ancestors is probably very few at each branch. The expansion of this gene family in seed plants and increased plant complexity seem to be correlated. As gradually the genomes of non-seed plants are becoming available it is worthwhile to gather the existing information about MADS box genes in non-seed plants. This review highlights various MIKC MADS box genes discovered so far in non-seed plants, their possible roles and an insight into their evolution.

## Introduction

The main principle of evo-devo (evolutionary developmental genetics) proposes that development and evolution are mutually interrelated processes (Gilbert et al., 1996). Since development is under genetic control, the genes which regulate the development of an organ may play a major role in their evolution (Theissen and Saedler, 1995). MADS-box gene family (*MCM1/AGAMOUS/DEFICIENS/SRF*) is one such important gene family which has been extensively explored because it is widely present in eukaryotes like plants, animals, fungi and might have originated at least 1 billion years ago (Purugganan et al., 1995; Theissen et al., 1996, 2000). MADS domain proteins are homeotic transcription factors with a DNA-binding MADS domain and play a major role during reproductive development (Schwarz-Sommer et al., 1990). They are broadly classified into two major classes based on their structure and phylogeny, Type I and Type II class (Alvarez-Buylla et al., 2000). Type I consists of Mα, Mβ, and Mγ subgroups that contain a MADS domain and a variable C terminal domain and Type II class genes have two subfamilies MIKC^C^ and MIKC^*^ that are characterized by a MADS domain, I region, K domain and C terminal domain (Ma et al., 1991; Henschel et al., 2002; Becker and Theissen, 2003). The I region may have a role in providing specificity to protein dimer formation, the K domain promotes protein dimerization and C terminal domain may function in transcriptional activation and in the formation of ternary and quaternary protein complexes (Ma et al., 1991; Davies and Schwarz-Sommer, 1994; Shore and Sharrocks, 1995; Riechmann and Meyerowitz, 1997; Cho et al., 1999; Egea-Cortines et al., 1999; Honma and Goto, 2001; Theissen and Saedler, 2001).

## MIKC type MADS-box genes in charophycean green algae

Charophycean green algae are the closest relatives of the extant land plants (Graham et al., 2000). Tanabe et al. (2005) isolated and characterized MADS-box genes from three Charophycean green algae, *CgMADS1* from the stonewort *Chara globularis, CsMADS1* from the coleochaete *Coleochaete scutata* and *CpMADS1* from the desmid *Clostridium peracerosum-strigosum-littorale* complex. The sequence analysis of these genes when compared with the already reported MIKC^C^ and MIKC^*^ type genes revealed that all the four M, I, K, and C domains were present in the isolated algal genes. Expression analysis suggests that these genes may have a putative role in haploid reproductive cell differentiation and during the course of evolution they were recruited into a diploid generation. The MIKC type genes in land plants fall into several groups with diverse functions and expression patterns, whereas in Charophycean green algae, only one MIKC^C^ type gene was reported in each of the three charophycean taxa (Tanabe et al., 2005). This extensive diversification of MIKC type genes may have played an important role in the development of sophisticated developmental systems in land plants (Tanabe et al., 2005).

## MIKC type MADS-box genes in bryophytes

Non-vascular land plants liverworts, mosses, and hornworts form the bryophyte group. According to current information the phylogeny of liverworts, mosses, and hornworts with respect to each other and its relation to land plants remains unresolved as there are multiple hypotheses for their positions (Szövényi, 2016).

Both Type I and Type II class of MADS-box genes have been reported from various bryophyte taxa. The pseudo-chromosomal genome assembly of the moss *Physcomitrella patens* confirmed the presence of 26 MADS-box genes. It has 6 MIKC^C^ type genes (*PPM1- 2, PpMADS1 and S, PPMC5, PPMC6*), 11 MIKC^*^ type genes (*PPM3-4, PPM6-7, PpMADS2-3, PPMA 8-12*), 2 pseudogenes (*PPMA5, PPTIM6*) and the rest are Type I (*PPTIM1-5,7,8*) where *PPTIM2,3* are Type I Mα genes and *PPTIM6-8* are Type I Mβ-γ genes (Krogan and Ashton, 2000; Henschel et al., 2002; Hohe et al., 2002; Riese et al., 2005; Quodt et al., 2007; Singer et al., 2007; Rensing et al., 2008; Singer and Ashton, 2009; Barker and Ashton, 2013, 2016).

Phylogenetic analysis reveals that MIKC^C^ genes from *P. patens* clustered into a separate clade from the MIKC^C^ type genes of other taxa suggesting that angiosperm orthologs are not found in mosses. The MIKC^*^ genes clustered within a larger cluster containing *Arabidopsis* MIKC^*^ genes. The Type I genes formed three clusters, one which was exclusively for *P. patens*, the second with Mα genes of *Arabidopsis* and third with Mβ-γ genes of *Arabidopsis* (Riese et al., 2005; Barker and Ashton, 2013). This suggests that at least 4 different types of MADS box genes existed in the most recent common ancestor (MRCA) of extant mosses and vascular plants about 450 million years ago (MYA) (Gramzow and Theissen, 2010; Barker and Ashton, 2013).

PPM1 and PpMADS1expression was detected in both vegetative and reproductive tissues in a study carried out using protein fused with GUS reporter. Though their expression pattern was broad, the protein was found at increased levels at the basal portions of archegonia and developing embryos, as well as in the seta and foot of sporophytes (Singer et al., 2007). Another protein localization study of PPM1 fused with reporters (GUS and citrine) expressed in both vegetative and reproductive tissues like chloronema, caulonema, tips of gametophores, gametophore apices, mature leaf cells, antheridia, and sporophytes (Koshimizu et al., 2018). PPM2 was reported to have a weak ubiquitous expression with increased expression in male and female gametangia and in the basal parts of the developing sporophytes (Quodt et al., 2007). This increased expression suggests that probably PPM2 has a role in defining the sink tissues for proper development of the organs required for transition from gametophyte to sporophyte (Quodt et al., 2007). In another study, PPM2 was found to be localized in different developmental stages like chloronema, caulonema, tips of gametophores, gametophore apices, mature leaf cells, antheridia, archegonia, and sporophytes. In the study by Quodt et al. (2007) PPM2 expression was detected in the basal part of the developing sporophyte whereas in the recent study by Koshimizu et al. (2018) expression is seen toward the apical part. It should be noted that this difference in expression pattern has not been discussed by Koshimizu et al. (2018). Another MIKC^C^ protein PPMC6 was also found to be localized in different tissues similar to PPM2 (Koshimizu et al., 2018). *PpMADS S* expression was found to be 3 fold higher in gametophore stage producing gametangia and sporophytes than gametophores without gametangia and sporophytes (Hohe et al., 2002). In localization studies carried out by Koshimizu et al. (2018) PpMADS S was detected in archegonia and at the base of antheridia but not in the older ones that had released their sperm. These studies suggest it may have a role during sexual reproduction of the moss (Hohe et al., 2002). PPMC5 localization was detected in chloronema, caulonema cells, throughout the gametophores, gametophore apices and mature leaf cells, archegonia, and sporophytes (Koshimizu et al., 2018). During sporophyte development, the spatiotemporal localization of the six proteins varied, with partial overlap (Koshimizu et al., 2018). These studies show that the MIKC^C^ genes may redundantly function in various moss developmental stages.

Deletion mutants and overexpression lines of these three MIKC^C^ genes (*PPM1, PPM2, PPMC6*) revealed that, these genes redundantly and negatively regulate the cell division and growth of the gametophore internodes thereby influencing the external water conduction which is necessary for the sperms to swim in order to reach and fertilize the eggs. They were also found to redundantly regulate the formation of motile flagella in sperms. Therefore, the authors suggest that both functions are necessary for fertilization to occur (Koshimizu et al., 2018). Earlier, the *PPM1* knockdowns created by antisense technology resulted in multifaceted mutant phenotype which may be due to the variation in the expressivity of the antisense *PPM1* gene or homology of the *PPM1* antisense RNA molecules to other *P. patens* MIKC^C^ genes (Singer et al., 2007). The knockdowns showed aberrant gametangia formation with significantly fewer antheridia and no archegonia. These lines also produced fewer sporophytes and sporophyte morphogenesis was abnormal as they were pale green, swollen and larger than control sporophytes (Singer et al., 2007). Single knock out mutants of MIKC^c^ genes in *Physcomitrella patens* were phenotypically normal when compared to the wildtype (Singer et al., 2007). The expression and functional studies in *P. patens* indicate that MIKC^C^ genes have broad expression pattern and play a role in both gametophyte and sporophyte generation.

Additionally in bryophytes MIKC^*^ genes are also present. Two MIKC^*^ type genes (*PPM6 and PPM7*) were isolated from *P. patens* (Riese et al., 2005). Zobell et al. (2010) isolated MIKC^*^genes from liverwort, *Marchantia polymorpha* (*MpMADS1)* and the mosses *Sphagnum subsecundum* (*SsMADS1-4*), and *Funaria hygrometrica* (*FhMADS1-11*). Their attempts to isolate MIKC^*^ genes from the hornworts *Anthoceros agrestis* and *Anthoceros formosae* was unsuccessful. The genes were either lost in the hornwort lineage or escaped identification due to unavailability of the whole genome (Zobell et al., 2010). But a recent article dealing with draft genome of *A. agrestis* mentions it has MADS box genes (details not mentioned) though the number is dramatically reduced when compared to *Physcomitrella* (Szövényi, 2016). It should be noted that completion of genome sequencing of *Anthoceros punctatus* will throw light on the status of MADS box genes in hornworts.

The liverwort gene *MpMADS1* was found to be the sister of all moss homologs thus suggesting that there was only one MIKC^*^ MADS-box gene before the separation of the major bryophyte lineages. *Sphagnum subsecundum* has fewer MIKC^*^ genes than *P. patens* and *F. hygrometrica* and formed a separate monophyletic clade suggesting expansion in the former was lesser in comparison to the latter. MIKC^*^ genes of *P. patens* and *F. hygrometrica* were grouped under one clade which is consistent as these two genera belong to the same family Funariales (Zobell et al., 2010).

Transcript level analysis showed ubiquitous expression of *PPM7* (MIKC^*^) with strongest signals in protonema and gametophores bearing sporophytes. *PPM6* (MIKC^*^) expression decreased from high in protonema to nearly undetectable in later stages of sporophytic development providing evidence for spatial and temporal regulation of this gene (Riese et al., 2005). MpMADS1 was found to be a functional transcription factor with the ability to bind to DNA and localize in the nucleus. The protein was also able to form homo-dimers. This gene was able to partially restore pollen germination in an *Arabidopsis* MIKC^*^ mutant (Zobell et al., 2010). *FhMADS* genes expression was strong in gametophytes and only residual in sporophytes. The expression was higher in protonema as compared to the gametophore indicating a role for *FhMADS* genes during vegetative development rather than promoting onset of reproductive development during the haploid phase (Zobell et al., 2010). The expression studies in *P. patens* and *F. hygrometrica* and functional study in *M. polymorpha* thus reveal that MIKC^*^ genes may play an important role during gametophyte development. Further studies to confirm the presence/absence of MIKC^C^ genes among these bryophyte species (*Funaria, Marchantia, and Sphagnum*) may be useful to understand the origin and evolution of MADS-box genes.

## MIKC type MADS-box genes in lycophytes

Lycophytes (clubmosses and spikemosses) are the most basal vascular plants and they appeared roughly 400 MYA (Kenrick and Crane, 1997). Surveying the MADS-box genes in these taxa may give us a better understanding of the evolutionary history of MADS-box genes and the evolution of advanced organs in flowering plants.

As seen in bryophytes, the lycophytes also have both Type I and Type II class MADS box genes. *Lycopodium annotinum* is a clubmoss in which 5 MIKC^C^ (*LAMB2-6*) MADS-box genes and 1 MIKC^*^ (*LAMB1*) MADS-box gene are reported so far (Svensson et al., 2000; Svensson and Engström, 2002). Tanabe et al. (2003) reported a MIKC^C^ type MADS-box gene, *SrMADS1* from the spike moss *Selaginella remotifolia*. Genome analysis of *Selaginella moellendorffii* revealed the presence of 19 putative MADS-box genes (Gramzow et al., 2012). It involves 13 type I genes (*SmMADS5-9, 11-15, 17-20*) where *SmMADS14,15* are of α type and SmMADS5,7,17 are of β-γ origin and rest are *Selaginella* type. This lycophyte also has 3 MIKC^C^ genes (*SmMADS1, 3, 6*), and 3 MIKC^*^ genes (*SmMADS 2, 4, 10*) (Gramzow et al., 2012).

Phylogenetic analysis showed that LAMB2-6 form a separate clade and does not show any orthology to moss and fern MIKC^C^ proteins (Svensson and Engström, 2002). SrMADS1 forms a separate clade with the LAMB2 group (LAMB2, 4, 6) in the phylogenetic analysis. So SrMADS1 may be sister to LAMB2 group (Tanabe et al., 2003). *SmMADS* MIKC^C^ were not monophyletic but *SrMADS1* and *SmMADS1* clustered together. These genes did not form a monophyletic group with *LAMB2,4,6* which was earlier reported by Tanabe et al. (2003) probably due to difficulties in determining the deep branching of widely divergent taxa (Gramzow et al., 2012). Notably no orthology was found between the MIKC^C^ of *S. moellendorffii* and angiosperms which suggests that the common ancestor of mosses and vascular plants had a single ancestral MIKC^C^ gene (Gramzow et al., 2012).

*LAMB2, 4* and *6* were similar to MADS-K-box genes and were grouped under MIKC^C^ class of genes. The other two genes (*LAMB3 and 5*) did not encode for a K domain. LAMB3 has only M, I domains and has a region with homology to partial K box which may be a truncated protein. LAMB5 has only M domain and ends 8 amino acid residues downstream of the M domain (Svensson and Engström, 2002). In SrMADS1, in addition to M, I, K, and C domains, SrMADS1 have additional amino acid residues in the N terminal of the MADS domain which was not reported in other genes of the LAMB2 group (Tanabe et al., 2003). *SmMADS1,3,6* contain the same number of exons as seen in *Arabidopsis* MIKC^C^ genes (Gramzow et al., 2012).

*LAMB2, 4, 5, 6* have broad expression patterns in sporophytic tissues such as roots and apices (Svensson and Engström, 2002). Expression of *SrMADS1* was found in all sporophytic tissues except in root and rhizophores. The expression pattern of *SmMADS1,3, 6* is currently not available. These findings hypothesize that MIKC^C^ genes may be involved in the development of sporophytic tissues like shoot, stem, sporogium in lycophytes (Tanabe et al., 2003).

Svensson et al. (2000) reported the first MIKC type gene *LAMB1* from *L. annotinum*. Although it has all the four domains – M, I, K, and C domains which is a prerequisite feature of classical MIKC genes, it differed from others in both sequence and structure as the intervening region is unusually longer and it also has a longer C region. These unusual characteristics of *LAMB1* made it different from the classical MIKC type genes (Svensson et al., 2000). *LAMB1* was thus hypothesized to be a primitive MIKC class gene, representing an ancestral state though they mentioned that the opposite possibility was also open (Svensson et al., 2000). In the following years after the discovery of MIKC^*^ class genes, phylogenetic analysis showed that LAMB1 clustered with the MIKC^*^ clade (Henschel et al., 2002; Gramzow et al., 2012; Kwantes et al., 2012). Thus *LAMB1* was the first MIKC^*^ gene to be discovered (Henschel et al., 2002). In the other lycophyte studied, S. *moellendorffii*, there are three MIKC^*^ genes namely *SmMADS2, 4, 10* which has similar exon-intron structure to that of *Arabidopsis* MIKC^*^ (Gramzow et al., 2012; Kwantes et al., 2012). Phylogenetic analysis revealed that the relationship of MIKC^*^ proteins of the lycophyte *S. moellendorffii* and LAMB1 of *L. annotinum* to the other clades of MIKC^*^ proteins or amongst themselves remained unresolved (Kwantes et al., 2012).

*LAMB1* expression was restricted to the reproductive structure strobili found in sporophyte during sporogenesis (Svensson et al., 2000). *SmMADS2, 4, 10* was shown to be expressed in microsporangia which have the male gametophyte- containing microspores (Kwantes et al., 2012). Thus in *Selaginella* evidence for strong gametophytic expression was shown whereas in *Lycopodium* only sporophytic expression was found. This shows that MIKC^*^ genes were also recruited in the sporophytes in early vascular plants but they did not have a conserved function in this generation (Kwantes et al., 2012).

## MIKC type MADS-box genes in ferns

Ferns are non-seed vascular plants with simple reproductive structures and form spores in the naked sporangium present on the abaxial side of the leaf (Gifford and Foster, 1989; Stewart and Rothwell, 1993; Hasebe et al., 1998).

The role of MADS box genes in the development of simple reproductive structures was looked into where, 2 MIKC^C^ genes (*CRM1, CRM3*) from the leptosporangiate fern, *Ceratopteris richardii* and 4 MIKC^C^ genes (*CRM2, CRM4, CRM5, CRM6*) from *Ceratopteris pteroides* was reported (Münster et al., 1997). Five MADS-box genes (*CMADS1-4*, and *6*) were isolated from *Ceratopteris richardii* by Hasebe et al. (1998) in which *CMADS3* and *CMADS6* were identical to the previously reported *CRM1* and *CRM3* genes. Münster et al. (1997) also isolated a MIKC^C^ gene (*OPM1*) from *Ophioglossum pendunculosum*, a eusporangiate fern thereby showing that MADS-box genes are present in other groups of ferns as well (Münster et al., 1997). They followed it up by discovering five cDNAs belonging to MIKC^C^ type MADS-box *OPM1-OPM5*, where *OPM1* and *OPM2* may represent closely related genes or alleles (Münster et al., 2002). In *Dryopteris*, a new MIKC^C^ MADS box gene *DfMADS1* was isolated which shared homology to other pteridophyte MADS-box genes (Huang et al., 2014). Thirty six putative MADS-box genes were reported in the endangered fern, *Vandenboschia speciosa* by the analysis of Next Generation Sequencing assembled transcriptome data. Among the reported 36 putative MADS-box genes, 1 gene was found to be type I, 32 were MIKC^C^ and 3 were MIKC^*^ type (Ruiz-Estévez et al., 2017).

Phylogenetic reconstruction resulted in 3 subfamilies of CRM proteins (CRM1-, CRM6-, and CRM3-like sequences) interspersed among spermatophyte clades (Münster et al., 1997). CMADS1, CMADS2/3/4, and CMADS6 fall under CRM6, CRM1, and CRM3 groups respectively (Hasebe et al., 1998). OPM1/2 and OPM5 may belong to the CRM6 family whereas the other proteins did not group into any specific clade of either the seed plants or the ferns (Münster et al., 2002). DfMADS1 groups with the CRM1 family of fern MADS domain proteins (Huang et al., 2014). VsMB2, 5, 7 proteins group with CRM1 subfamily and VsMB3 and VsMB6 proteins groups with the CRM3 subfamily and VsMB4 to the CRM6-like subfamily (Ruiz-Estévez et al., 2017). The results discussed here show that the leptosporangiate ferns studied till now have genes representing three clades *CRM1, CRM3*, and *CRM6* whereas *Ophioglossum* has three *CRM6* like genes along with 2 unique genes which may be characteristic to the eusporangiate ferns. Further work on eusporangiate ferns will be required to validate the origin of these unique genes and whether these genes are specific to eusporangiate ferns. In the phylogenetic trees discussed, Münster et al. (1997, 2002) show that CRM3 clade is closer to the AG clade whereas Hasebe et al. (1998) and Huang et al. (2014) show that CRM6 clade is closely related to the AG clade. The latter trees seem to be clearer since the CRM6 proteins (CMADS1, CerMADS2, CerMADS3) have additional N terminal amino acids preceding the MADS domain which is similar to the AG protein. Another variation noticeable is that, according to Hasebe et al. (1998) CRM3 and CRM6 clades are closely associated whereas in all the other studies CRM1 and CRM3 are phylogenetically close. Thus the position of CRM3 within the fern clades remains unclear but CRM1 and CRM6 always formed distinct clades. Hence according to the studies discussed, the MRCA of ferns and seed plants probably had at least 2 MIKC^C^ type genes. Further gene duplication events might have led to lineage specific diversification and expansion of MIKC^C^ class in extant ferns and seed plants.

Northern blot analysis showed *CRM1 and CRM3* were expressed in the gametophytic (haploid) as well as the sporophytic (diploid) phase of the fern life cycle (Münster et al., 1997). *CMADS1 (CRM6), CMADS 2-4* (*CRM1*) expression was detected in both vegetative and reproductive tissues of the sporophyte and *CMADS6* (*CRM3*) was detected in gametophytic tissues but not in sporophytic tissues. Further spatiotemporal survey of mRNA expression of these MADS genes has revealed that they may be involved in regulating cell division during early organ development and playing an unknown role in the differentiated vasculature(Hasebe et al., 1998). *OPM1, OPM3, OPM5* were expressed in both trophophore and sporophore at an almost same level of expression, whereas *OPM4* was detected only in sporophore. Thus this indicated that *OPM4* has a role specific to spore development whereas the other genes may have a universal role. The ubiquitous expression of *OPM1, OPM3*, and *OPM5* are similar to the genes of *Ceratopteris*, which is characteristic to the MADS-box genes of the fern family but not an absolute feature (Münster et al., 2002). Though *DfMADS1* was expressed in both sporophytes and gametophyte, it was expressed at very high levels in the spores and in the young prothallus indicating that this particular MADS-box gene may be involved in spore germination and reproductive development (Huang et al., 2014). In *V. speciosa*, the expression level of 6 MIKC^C^ type MADS-box genes (*VsMB2, VsMB3, VsMB4, VsMB5, VsMB6, VsMB7*) was analyzed in sporophytes, gametophytes and sporangia. All the six genes (*VsMB2-7*) and most of the genes (*VsMB3, VsMB5, VsMB6, VsMB7*) were found to have a broad expression pattern in sporophytes and gametophytes respectively. *VsMB2* and *VsMB4* expression level in gametophytes was found to be residual when compared to their expression level in sporophytes. These genes may have a specialized role in the changes occurring during the alternation of the two major phases in the life cycle of *V. speciosa*. Also, reduced expression of *VsMB3 and VsMB7* genes was reported in the sporangium which is a reproductive structure. The down-regulation of these genes may be important for the development of the reproductive structure, sporangia (Ruiz-Estévez et al., 2017).

Four MIKC^*^ type MADS-box genes namely *CRM13-16* were reported from *Ceratopteris richardii* (Kwantes et al., 2012). Phylogenetic analysis showed that, CRM13 and CRM16 grouped into the P clade whereas CRM14 and CRM15 into the S clade thus indicating that there were two different types of MIKC^*^ genes in the ancestor of ferns and spermatophytes (Kwantes et al., 2012). Expression analysis revealed high expression of all the genes in roots and both S-clade (*CRM14*) and P-clade (*CRM16*) genes in male and hermaphroditic gametophytes. In other tissues such as fertile and unfertile blades, even though the S-clade genes expressed the expression of P-clade genes were found to be dominating (Kwantes et al., 2012). P-clade members formed homodimers and heterodimers which might function in both sporophytic and gametophytic tissues, but heterodimers between the members of the S-(CRM14) and P-clades (CRM16) were shown to be typical for the gametophytes and roots of *Ceratopteris* (Kwantes et al., 2012).

## Evolution of MADS box genes in non-seed plants

**Figure 1:**
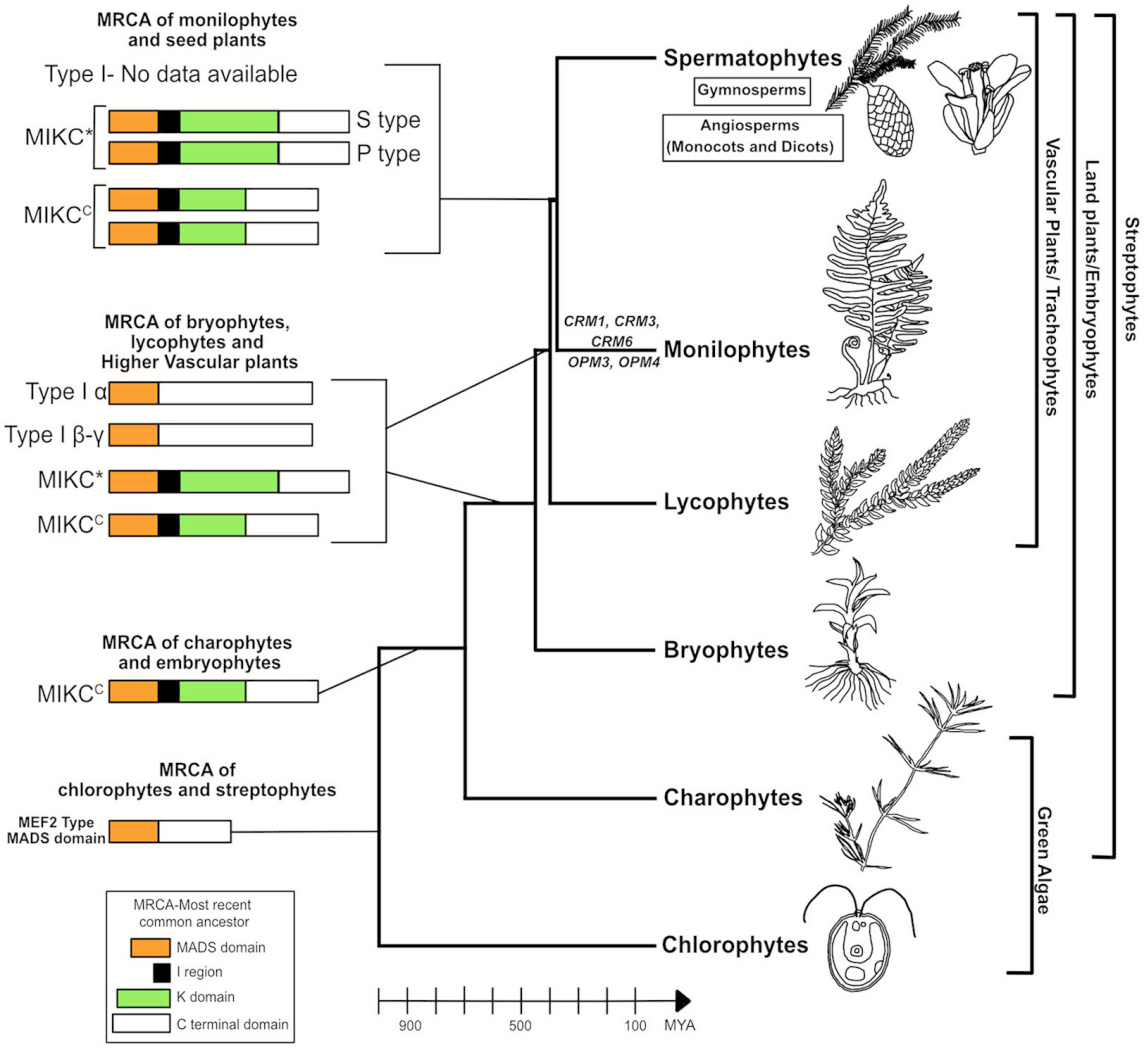
Model for evolution of MADS box genes in non-seed plants. MIKC-type MADS proteins seem to have evolved in streptophytes (700 MYA) by addition of K domain from a MEF2-like ancestor in chlorophytes. A gene duplication event in the common ancestor of bryophytes and tracheophytes (450 MYA) led to MIKC^C^ and MIKC* proteins where MIKC*-type proteins have an elongated K domain. The origin of Type I is still not clear which lacks the I region and K domain but in the MRCA of bryophytes, lycophytes and tracheophytes there are two subtypes M alpha and M beta-gamma. The information on Type I genes in the MRCA of monilophytes and spermatophytes is not available. The MRCA of monilophytes/ferns and seed plants (380 MYA) had at least 2 MIKC^C^ type and 2 MIKC* type (S and P clades) genes. Further diversification and expansion took place independently in MIKC^C^ type genes in the lineages leading to extant ferns and spermatophytes. This gave rise to the large number of MIKC^C^ genes in present day ferns and seed plants. In leptosporangiate ferns, there are genes belonging to three clades *CRM1, CRM3*, and *CRM6* whereas in *Ophioglossum* (a eusporangiate fern) in addition to the three *CRM6*-like genes there are two unique genes *OPM3* and *OPM4* which may be specific to eusporangiate ferns. It should also be noted that in each taxa from bryophytes onwards there has been lineage specific diversification and expansion of MIKC genes. It is a possibility that with increase in number of MIKC genes there has been increase in complexity (from single celled algae to multicellular algae to multicellular non-vascular plants to early vascular plants and finally seed plants) which needs further evidence. MYA, million years ago.

Genome analysis of the green algae *Chlamydomonas reinhardtii* (Tanabe et al., 2005), *Ostreococcus tauri* (Derelle et al., 2006), *Ostreococcus lucimarinus* (Palenik et al., 2007) and the red algae *Cyanidioschyzon merolae* (Matsuzaki et al., 2004) reported no MIKC^C^ or MIKC^*^ type genes although a single gene with MADS box lacking I,K,C region in *C. reinhardtii* and *C. merolae* and lacking K domain in *Ostreococcus sp's* was found which resembled the MEF2 type MADS domain (Type II like) (Kaufmann et al., 2005; Tanabe et al., 2005). Hence the MRCA of chlorophytes and streptophytes contained a protein with MADS domain similar to Type II approximately 1,000 million years ago (MYA) representing the ancestral MADS domain protein (Kaufmann et al., 2005). One MIKC^C^ type MADS-box gene was reported in each of the three Charophycean green algal species, which are representatives of charophytes that are believed to be the common ancestor of land plants (Tanabe et al., 2005). These findings suggest that the MIKC type MADS-box genes evolved by the addition of a K domain to the ancestral MADS-box gene in the charophycean—land plant lineage after its divergence from the *Chlamydomonas* lineage at least 700 MYA (Figure 1) (Kaufmann et al., 2005; Tanabe et al., 2005; Gramzow and Theissen, 2010). There were at least 2 types of ancestral MIKC genes (1 MIKC^C^ and 1 MIKC^*^) and 2 types of Type I genes (α and β-γ) in the common ancestor leading to bryophytes (450 MYA), lycophytes (400 MYA) and higher vascular plants (Figure 1) (Gramzow and Theissen, 2010; Gramzow et al., 2012; Barker and Ashton, 2013). It is in the common ancestor of bryophytes and vascular plants that MIKC diverged into MIKC^C^ and MIKC^*^, no further diversification seems to have taken place in lineage leading to lycophytes (Figure 1)(Gramzow et al., 2012; Barker and Ashton, 2013). The MRCA of monilophytes/ferns and seed plants (380 MYA) had at least 2 MIKC^C^ type and 2 MIKC^*^ type (S and P-type) genes (Figure 1) (Münster et al., 1997, 2002; Hasebe et al., 1998; Huang et al., 2014; Ruiz-Estévez et al., 2017). The information on Type I genes in ferns is currently very limited, only 1 gene reported in *V. speciosa* (Ruiz-Estévez et al., 2017). In each of the taxa (bryophytes, lycophytes, and ferns) discussed, there has also been lineage specific diversification and expansion of the MADS box genes which have led to the unique body plan of these non-seed plants (Gramzow et al., 2012; Barker and Ashton, 2013; Ruiz-Estévez et al., 2017). Thus it can be hypothesized that the diversification and duplication of Type II MIKC genes accelerated in the common ancestor of monilophytes and seed plants and continued to expand extensively in the spermatophytes.

Structurally the MIKC genes of non-seed plants and seed plants are very similar and the proteins function in a similar mode by forming homodimers and heterodimers as inferred from the reports available for MIKC^*^ proteins (Tanabe et al., 2005; Zobell et al., 2010; Kwantes et al., 2012). Some of the CRM6 clade proteins (CMADS1, CerMADS2, and CerMADS3) have additional amino-terminal amino acids like AGAMOUS (AG), they seem to be the most closely related to the AG clade in the seed plants than other MADS box genes (Hasebe et al., 1998). One MIKC^C^ gene from *S. remotifolia* (*SrMADS1*), one MIKC^C^ (*SmMADS1*) and four Type I genes (*SmMADS12,18,19,20*) from *S. moellendorffii* and all the MIKC^C^ genes from *P.patens* also have an N-terminal region preceding the MADS box (Tanabe et al., 2003; Gramzow et al., 2012; Barker and Ashton, 2013). This shows that the N-terminal region preceding the MADS box region was retained in *AGAMOUS* genes as well as some other genes in extant seed plants (Mizukami et al., 1996). It has been shown that this region in AG is not necessary for DNA binding *in-vitro* (Mizukami et al., 1996). Unlike AG, the N terminal region of SRF has been shown to be involved in DNA binding and affinity *in-vitro* (Sharrocks et al., 1993; Nurrish and Treisman, 1995), hence the function of the N-terminal region in MADS domain proteins of non-seed plants remains to be explored.

## Origin of MIKC^*^ type MADS-box genes

Henschel et al. (2002) proposed two hypotheses regarding the difference between MIKC^*^ and MIKC^C^ genes. According to the authors there were differences in the length of I domain and K domain between MIKC^*^ and MIKC^C^ genes. According to the first hypothesis during evolution the ancestral I domain might have elongated in the MIKC^*^ genes. The second hypothesis states that the difference in length upstream of C domain between MIKC^C^ and MIKC^*^ type genes is due to de novo insertion within K domain and not due to elongation of I domain. Sequence analysis and exon-intron structure analysis of various MIKC-type genes favored longer I domain hypothesis wherein the I region of MIKC^*^ was found to be encoded by 4 or 5 exons whereas in MIKC^C^ it is encoded by just one exon (Henschel et al., 2002). Later Kwantes et al. (2012) redefined the existing hypotheses which seems more reliable that states, MIKC^C^ type gene was found in the common ancestor of charophycean-land plant lineage and later duplication events in the first part of the Keratin-like region resulted in the evolution of MIKC^*^ group genes which distinguished it from classical MIKC^C^ type gene (Kwantes et al., 2012).

Some MIKC^C^ gene orthologs of angiosperms are reported in gymnosperms with functional conservation (Sundstrom and Engstrom, 2002). But no such orthologs are so far identified in non-seed plants (Henschel et al., 2002). This suggests MIKC^C^ class of genes have undergone independent gene duplications and functional diversifications during evolution which resulted in the extant highly diversified MIKC^C^ type MADS-box genes in seed plants (Henschel et al., 2002). This may be a probable reason for the absence of traces of orthology of floral MIKC^C^ type genes in non-seed plants (Henschel et al., 2002). In contrast, MIKC^*^ genes show considerable orthology in ferns, gymnosperms, angiosperms and branched into two monophyletic subgroups S and P (Nam et al., 2004; Verelst et al., 2007; Gramzow et al., 2012; Kwantes et al., 2012; Liu et al., 2013). It should be noted that the MIKC^*^ gene family has a small size in plants across taxa and the S and P clades have been the only reported diversification in the monilophytes and spermatophytes. It is thus hypothesized that conservation of these clades reveals a key role that has been maintained highly similar during the evolution of these plant groups (Kwantes et al., 2012).

## Is size of MIKC MADS-box gene family and complexity of the organism interrelated?

There is an existing hypothesis that size of a gene family involved in development is correlated to the complexity of the organism (Floyd and Bowman, 2007; Quodt et al., 2007). The size of MIKC type MADS-box gene family varies from one in algae, 17 in *Physcomitrella* to 37 in Rice and 46 in *Arabidopsis* (Parenicova et al., 2003; Tanabe et al., 2005; Arora et al., 2007; Barker and Ashton, 2013, 2016). This suggests a trend of increasing MIKC numbers during evolution of green plants which implies a correlation between surge in gene numbers and developmental complexity (single celled algae to multicellular algae to multicellular non-vascular plants to early vascular plants and finally seed plants) (Quodt et al., 2007). One such example of the above hypothesis with respect to the MADS box gene family is of the MIKC^*^ clade in bryophytes where the authors speculate that increase in MIKC^*^ genes is related to evolutionary change in growth habit (Zobell et al., 2010). The authors predict that the growth pattern changes from an ancestral predominantly thalloid growth (one gene in *M. polymopha*) to a condition of transiently thalloid but mainly leafy growth (four or more genes in *S subsecundum*) or a condition of both filamentous and leafy growth (11 genes in *F. hygrometrica* and *P. patens*), can be assigned to increase in gene numbers, however further studies will be required to substantiate this hypothesis (Zobell et al., 2010). Thus it is predicted that MIKC type MADS-box gene family diversified and duplicated extensively after the separation from the charophycean lineage and expansion of this gene family was important for the evolution of advanced morphological features in extant seed plants.

## Role of MIKC MADS-box genes in non-seed plants

In flowering plants, MIKC^C^ and MIKC^*^ genes have more specific expression patterns in sporophytes and gametophytes and its functions are well studied; refer to **Table 1D**. This specialized expression probably evolved with increase in complexity of the organs in extant seed plants (Münster et al., 1997; Theissen et al., 2000; Theissen and Saedler, 2001; Gramzow and Theissen, 2010). The literature available in non-seed plants, mostly reports the broad expression pattern of MIKC^C^ genes in both sporophytes and gametophytes; refer to **Table 1B** (Münster et al., 1997, 2002; Hasebe et al., 1998; Huang et al., 2014). This has led to the hypothesis that non-seed plant MIKC^C^ genes may not have any organ specific roles; instead it may have a generalized role in the transcriptional control of different developmental events during its life cycle (Münster et al., 1997; Hasebe et al., 1998; Huang et al., 2014). However there are exceptions reported in some studies which cannot be ignored. Few MIKC^C^ genes were found to have specific expression pattern rather than ubiquitous expression (Münster et al., 2002; Tanabe et al., 2005; Huang et al., 2014; Ruiz-Estévez et al., 2017). *DfMADS1* gene was proposed to have role in spore germination and reproductive development of *Dryopteris fragrans* due to its high expression in spores and prothallus (Huang et al., 2014). *VsMB2, VsMB4* may be important in the alternation of the two main phases of *V. speciosa* life cycle as it is specifically down regulated in the gametophytes (Ruiz-Estévez et al., 2017). *OPM4* gene of *O. pedunculosum* expressed exclusively in the reproductive structures (Münster et al., 2002) while *VsMB3, VsMB7* were reported to have reduced expression in the reproductive structures of *V. speciosa* (Ruiz-Estévez et al., 2017) which shows that differential expression of these genes with respect to the vegetative structures may be critical for the development of the reproductive structures. The MIKC^C^ genes function in the formation of motile flagella sperms in *Physcomitrella patens* (Koshimizu et al., 2018) and have a putative role in flagellate sperm differentiation in *Chara globularis* (Tanabe et al., 2005) suggesting they regulate flagellum-related genes in mosses and charophycean green algae. The absence of these homologs in *Arabidopsis* suggests that they may have been lost during transition from the use of flagellate sperm to pollen tubes for fertilization (Koshimizu et al., 2018). Another function of the MIKC^C^ genes from *P. patens* seems to be its involvement in gametangia formation i.e., differentiation of reproductive structures from the non-reproductive tissues in the gametophore. This function is analogous to the class C genes in spermatophytes and thus may represent an ancestral function which remained conserved through evolution though the genes have not been shown to be orthologous (Quodt et al., 2007; Singer et al., 2007; Singer and Ashton, 2009).

From the studies discussed above in bryophytes, lycophytes and ferns it seems MIKC^*^ genes have a conserved expression and function with respect to haploid phase of the life cycle (Svensson et al., 2000; Henschel et al., 2002; Zobell et al., 2010; Kwantes et al., 2012). The higher proportion of MIKC^*^ genes than MIKC^C^ genes in bryophytes as compared to vascular plants (Tables 1A,D) indicates that these genes have a role to play in a gametophyte -dominant life cycle (Gramzow et al., 2012). Functional conservation of MIKC^*^ genes across taxa was demonstrated by the partial rescue of pollen *in vitro* germination defect of *Arabidopsis* MIKC^*^ mutant pollen by MpMADS1, an MIKC^*^ protein from *Marchantia* (Zobell et al., 2010; Kwantes et al., 2012). It is possible that perhaps with time their function was restricted to the male gametophyte in the lineage leading to monocots and higher eudicots about 150 million years ago (Verelst et al., 2007; Liu et al., 2013). This kind of conservation has not been reported for MIKC^C^ transcription factors thus suggesting a conserved role of MIKC^*^ transcription factors during gametophytic development (Zobell et al., 2010; Kwantes et al., 2012).

**Table 1A:** List of non-seed plants containing MADS-box genes- type and number.

	**Type I**	**MIKC^C^**	MIKC*	**Total**	**References**
**ALGAE**					
*Chara globularis*	NR	1	NR	1	Tanabe et al., 2005
*Coleochaete scutata*	NR	1	NR	1	Tanabe et al., 2005
*Clostridium peracerosum*	NR	1	NR	1	Tanabe et al., 2005
**BRYOPHYTES**					
**Liverworts**					
*Marchantia polymorpha*	NR	NR	1	1	Zobell et al., 2010
**Mosses**					
*Physcomitrella patens* (including pseudogenes)	8	6	12	26	Rensing et al., 2008; Barker and Ashton, 2013
*Funaria hygrometrica*	NR	NR	11	11	Zobell et al., 2010
*Sphagnum subsecundum*	NR	NR	4	4	Zobell et al., 2010
**PTERIDOPHYTES**					
**Lycophytes**					
*Lycopodium annotinum*	NR	5	1	6	Svensson et al., 2000; Svensson and Engström, 2002
*Selaginella remotifolia*	NR	1	NR	1	Tanabe et al., 2003
*Selaginella moellendorffii*	13	3	3	19	Gramzow et al., 2012
*Selaginella pallescens*	NR	NR	3	3	Kwantes et al., 2012
**Ferns**					
*Ceratopteris richardii*	NR	5	4	9	Münster et al., 1997; Hasebe et al., 1998; Kwantes et al., 2012
*Ceratopteris pteroides*	NR	5	NR	5	Münster et al., 1997
*Ophioglossum pendunculosum*	NR	5	NR	5	Münster et al., 1997, 2002
*Dryopteris fragrans* (L.) Schott	NR	1	NR	1	Huang et al., 2014
*Vandenboschia speciosa*	1	32	3	36	Ruiz-Estévez et al., 2017

*NR, not reported*.

**Table 1B:** General expression pattern of MADS-box genes in non-seed plants.

**Non-seed plants with MADS box genes**	**Type**	**General expression pattern**		**References**
		**Type of generation**	**Type of tissue**	
**ALGAE**				
*Chara globularis*	MIKC^C^	Gametophytes	Oogonium and antheridium during differentiation	Tanabe et al., 2005
*Clostridium peracerosum*	MIKC^C^	Gametophytes	Gametangial cells	Tanabe et al., 2005
**BRYOPHYTES**				
**Moss**				
*Physcomitrella patens*	MIKC^C^	Gametophyte and sporophyte	Vegetative and reproductive tissues PPM1 higher in basal portions of archegonia and developing embryos, as well as in the seta and foot of sporophytes, chloronema, caulonema, tips of gametophores, gametophore apices, mature leaf cells, antheridia PpMADS1 higher in basal portions of archegonia and developing embryos, as well as in the seta and foot of sporophytes PPM2, PPMC6 sporophytes, chloronema, caulonema, tips of gametophores, gametophore apices, mature leaf cells, antheridia, archegonia PpMADS1 3 fold higher in gametophore stage producing gametangia and sporophytes than gametophores without gametangia and sporophytes, archegonia and at the base of antheridia but not in the older ones that had released their sperm PPMC5 chloronema, caulonema cells, throughout the gametophores, gametophore apices and mature leaf cells, archegonia and sporophytes During sporophyte development, the spatiotemporal localization of the six proteins varied, with partial overlap	Krogan and Ashton, 2000; Hohe et al., 2002; Quodt et al., 2007; Singer et al., 2007; Singer and Ashton, 2009; Koshimizu et al., 2018
*Physcomitrella patens*	MIKC*	Gametophyte and sporophyte	Vegetative and reproductive tissues *PPM6* high in protonema to nearly no detectable expression during later stages of development *PPM7* strongest signals in protonema and gametophores bearing sporophytes	Riese et al., 2005
*Funaria hygrometrica*	MIKC*	Strong expression in gametophytes and residual in sporophytes	Higher in protonemata (filamentous stage, vegetative stage) than in gametophores (leafy stage which bears the reproductive organs)	Zobell et al., 2010
**PTERIDOPHYTES**				
**Lycophytes**				
*Lycopodium annotinum* (Gametophytic expression is not reported)	MIKC^C^	Broad expression in sporophytes	Stronger expression in vegetative tissues than in the reproductive tissue, strobili	Svensson and Engström, 2002
*Lycopodium annotinum* (Gametophytic expression is not reported)	MIKC*	Sporophytes	Expressed exclusively in the reproductive tissue, strobili	Svensson et al., 2000; Svensson and Engström, 2002
*Selaginella remotifolia* (Gametophytic expression is not reported)	MIKC^C^	Broad expression in sporophytes except roots and rhizophores	Both in vegetative and reproductive structures such as vegetative stage, strobili, microphylls, stems	Tanabe et al., 2003
*Selaginella moellendorffii*	MIKC*	Sporophytes and gametophytes but high in male gametophyte	Vegetative and reproductive structures but high in microsporangia	Kwantes et al., 2012
*Selaginella pallescens*	MIKC*	Gametophytes	Reproductive structures (micro and megasporongia)	Kwantes et al., 2012
**Ferns**				
*Ceratopteris richardii*	MIKC^C^	Gametophytes and sporophytes. *CMADS6* was expressed only in gametophytes.	Vegetative and reproductive tissues of sporophyte	Münster et al., 1997; Hasebe et al., 1998
*Ceratopteris richardii*	MIKC*	Gametophytes and sporophytes	Vegetative and reproductive tissue of sporophytes (unfertile and fertile blades)	Kwantes et al., 2012
*Ophioglossum pendunculosum* (Gametophytic expression is not reported)	MIKC^C^	Sporophytes	Vegetative and reproductive tissue of sporophyte. *OPM4* is expressed only in reproductive tissue of sporophyte	Münster et al., 2002
*Dryopteris fragrans* (L.) Schott	MIKC^C^	Gametophytes and sporophytes	Vegetative and reproductive tissue but high during spore germination and reproductive development	Huang et al., 2014
*Vandenboschia speciosa*	MIKC^C^	Gametophytes and sporophytes (*VsMB2* and *VsMB4* had residual expression in gametophytes)	Vegetative and reproductive tissue (*VsMB3 and VsMB7* had reduced expression in sporangia)	Ruiz-Estévez et al., 2017

**Table 1C:** Role of individual MADS-box gene reported in non-seed plants.

**Species**	**Type**	**Gene**	**Function**	**References**
**ALGAE**				
*Chara globularis*	MIKC^C^	*CgMADS1*	Haploid reproductive cell differentiation^†^	Tanabe et al., 2005
*Clostridium peracerosum*	MIKC^C^	*CpMADS1*	Haploid reproductive cell differentiation^†^	Tanabe et al., 2005
**BRYOPHYTES**				
**Moss**				
*Physcomitrella patens*	MIKC^C^	*PpMADS-S*	Sexual reproduction^†^	Hohe et al., 2002
*Physcomitrella patens*	MIKC^C^	*PPM2*	Nutrient supply and the development of sink tissues^†^	Quodt et al., 2007
*Physcomitrella patens*	MIKC^C^	*PPM1, PPM2, PpMADS1*	Diverse aspects in developmental program, including gametangia formation, sporophyte development and leaf morphogenesis^*^	Singer et al., 2007
*Physcomitrella patens*	MIKC^C^	*PPM1, PPM2, PPMC6*	Cell division and growth of gametophore internodes; formation of motile flagella in sperms^*^	Koshimizu et al., 2018
**PTERIDOPHYTES**				
**Lycophytes**				
*Selaginella remotifolia*	MIKC^C^	*SrMADS1*	Development of basic sporophytic tissues such as shoot, stem, and sporangium^†^	Tanabe et al., 2003
*Selaginella moellendorffii*	MIKC^*^	*SmMADS2, SmMADS4, SmMADS10*	Function in gametophytes^†^	Kwantes et al., 2012
*Selaginella pallescens*	MIKC^*^	*SpMADS1, SpMADS2, SpMADS3*	Function in gametophytes^†^	Kwantes et al., 2012
**Ferns**				
*Dryopteris fragrans* (L.) Schott	MIKC^C^	*DfMADS1*	Spore germination and reproductive development^†^	Huang et al., 2014
*Ophioglossum pendunculosum*	MIKC^C^	*OPM4*	Role in reproduction^†^	Münster et al., 2002
*Vandenboschia speciosa*	MIKC^C^	*VsMB2, VsMB4*	Regulate changes in the alternation of two generations^†^	Ruiz-Estévez et al., 2017
*Vandenboschia speciosa*	MIKC^C^	*VsMB3, VsMB7*	Development of sporangia^†^	Ruiz-Estévez et al., 2017

* *Function determined based on knock-outs and knock-downs*.

† *putative function assigned based on expression pattern*.

**Table 1D:** Type, number and role of MADS box genes in Angiosperms.

**MADS-box class**	**Important roles in angiosperms**			**References**
MIKC^C^ genes	Control floral organogenesis			Coen and Meyerowitz, 1991
	Floral meristem development, floral transition, senescence and abscission of flowers, embryonic development			Fernandez et al., 2000
	Leaf and root morphogenesis			Tapia-Lopez et al., 2008
	Nodulation			Heard and Dunn, 1995; Heard et al., 1997; Zucchero et al., 2001
	Fruit development and dehiscence			Rijpkema et al., 2007
MIKC* genes	Pollen development, embryogenesis, early seedling development, silique development			Kofuji et al., 2003; de Folter et al., 2004; Lehti-Shiu et al., 2005; Verelst et al., 2007
**No. of genes reported**	**Type I**	**Type II MIKC**^C^	Type II MIKC*	
*Oryza sativa*	38	32	5	Arora et al., 2007
*Arabidopsis thaliana*	61	39	7	Parenicova et al., 2003

## Future prospects

These limited reports on the specific roles of MIKC genes in non-seed plants are noteworthy. It gives us clues that non-seed plant MIKC genes may also have targeted roles. It questions the existing hypothesis on the generalized function of the non-seed plant MIKC genes. Extensive research has not yet been carried out in non-seed plants as most of the functions assigned to the non-seed plant MIKC genes are putative and on the basis of expression studies (Refer to Table 1C). Knock-outs and overexpression studies in near future to study the exact role of these genes may help us to come up with a clearer picture about the role of MIKC genes in non-seed plants. An initial insight on the hornwort species, *Anthoceros agrestis* has given a hint that there was a dramatic reduction in the MADS-box gene family size when compared to that of *P. patens* (Szövényi, 2016). A detailed report on the MADS-box genes in hornworts is still lacking. Hornwort shares some common characteristics with both algae and spermatophytes and is believed to be the immediate sister for all the tracheophytes (vascular plants) (Renzaglia, 1978). Exploring hornwort lineage will be very essential to know the role and evolution of MADS-box gene family in land plants. Information on the MIKC^C^ genes in *Marchantia, Funaria*, and *Sphagnum* is also missing hence further work will be required to bring to light their presence or absence in these bryophyte species (Zobell et al., 2010). Like *AGAMOUS*, some MIKC^C^ genes from *Ceratopteris, Selaginella*, and *Physcomitrella* have an N-terminal region preceding the MADS-box, the importance of this region remains to be explored in non-seed plants. Extending functional studies like mutational analysis, gene silencing and targeted genome editing using the CRISPR/Cas9 system to non-seed plants in near future may be useful in understanding the ancestral function of MADS-box genes. Acquiring knowledge on functional and structural aspects of MADS-box gene family in non-seed plants may give us clues on the evolution of advanced organs and developmental processes in extant land plants.

## Authors contributions

SN conceived the idea; GT and SN conceptualized and drafted the review; SN finalized the review.

### Conflict of interest statement

The authors declare that the research was conducted in the absence of any commercial or financial relationships that could be construed as a potential conflict of interest.
